# Neurofeedback training: Decreases in Mu rhythm lead to improved motor performance in complex visuomotor skills

**DOI:** 10.1007/s12144-022-03190-z

**Published:** 2022-05-18

**Authors:** Kuo-Pin Wang, Cornelia Frank, Tsung-Min Hung, Thomas Schack

**Affiliations:** 1 Center for Cognitive Interaction Technology (CITEC), Bielefeld University, Inspiration 1, 33619 Bielefeld, Germany; 2 Neurocognition and Action - Biomechanics Research Group, Faculty of Psychology and Sports Science, Bielefeld University, Universitätsstraße 25, 33615 Bielefeld, Germany; 3 Sports and Movement Group, Department of Sports Science, School of Educational and Cultural Studies, Osnabrück University, Jahnstraße 75, 49080 Osnabrück, Germany; 4 Department of Physical Education and Sport Sciences, National Taiwan Normal University, No. 162, Section 1, Heping East Road, Da-an District, Taipei, 106 Republic of China (Taiwan); 5 Institute for Research Excellence in Learning Science, National Taiwan Normal University, No. 162, Section 1, Heping East Road, Da-an District, Taipei, 106 Republic of China (Taiwan)

**Keywords:** Golf, Shooting, Implicit motor learning, Simple motor skills, Complex motor skills

## Abstract

The physiological function of the Mu rhythm (8–13 Hz in the central region) is still unclear, particularly its role in visuomotor performance in sports (shooting vs. golf putting), as both the complexity of the motor skills (i.e., simple vs. complex visuomotor skills) and the skill level (e.g., novices vs. experts or low-skilled vs. highly skilled) may modulate Mu rhythm. To gain a broader understanding of the association between Mu rhythm and visuomotor skill performance, a study design that considers both a control moderator (the difference in skill level) and the ability to manipulate Mu rhythm (i.e., either increase or decrease Mu rhythm) is required. To achieve this, we recruited 30 novice golfers who were randomly assigned to either the increased Mu rhythm group (IMG), decreased Mu rhythm group (DMG), or sham group (SG) and used electroencephalographic-neurofeedback training (EEG-NFT) to manipulate Mu rhythm during a golf putting task (complex visuomotor skill). The aim was to determine whether the complexity of the motor skill was a potential moderator of Mu rhythm. We mainly found that Mu power was significantly decreased in the DMG following EEG-NFT, which lead to increased motor control and improved performance. We suggest that (1) the complexity of the motor skill, rather than the difference in skill level, may be a potential moderator of Mu rhythm and visuomotor performance, as our results were not consistent with a previous study that reported that increased Mu rhythm improved shooting performance (a simple visuomotor task) in novices.

In precision sports, maintaining an optimal psychological state during the pre-performance period is vital to achieving optimal performance (Bortoli et al., 2012). To achieve an optimal psychological state in precision sports such as golf putting or shooting motor programming is an essential psychological feature. Motor programming processes the representation of the skill that organizes and controls the many degrees of freedom involved in performing an action (Schmidt et al., 2018). Motor programming allows athletes to exert the appropriate motor control (i.e., movement force, direction, or stability) for a superior performance (Cooke et al., 2014; di Fronso et al., 2016; Wang et al., 2019). Given that superior motor performance depends significantly on the regulation of motor programming processes during the execution of a skill (Bertollo et al., 2016; Cooke et al., 2015), identifying novel ways to refine motor programming processes is crucial for sport performance enhancement.

Previous electroencephalogram (EEG) studies have shown that motor programming processes are associated with the sensorimotor Mu rhythm (8–13 Hz in the central region) during motor preparation (Cooke et al., 2014, 2015). Specifically, the Mu rhythm reflects the allocation of cognitive resources allocation to response motor programming (Cooke et al., 2015) during the observation and execution phases of goal-directed actions (Cannon et al., 2014). For example, Denis et al. (2017) showed that reduced Mu rhythm in the central region (i.e., activation of the central area) is associated with successful movement identification. Similarly, in a meta-analysis, Fox et al. (2016) showed that Mu rhythm was significantly decreased in the central region during the execution (Cohen’s *d* = 0.46, N = 701) and observation (Cohen’s *d* = 0.31, N = 1,508) phases of an action. In the field of sport psychophysiology, Mu activity influences visuomotor performance during golf putting (Babiloni et al., 2008; Cooke et al., 2014; Wang et al., 2019, 2020) and shooting (Bertollo et al., 2016; Del Percio et al., 2009; Haufler et al., 2000).

Although Mu rhythm is associated with motor programming processes, the physiological function of the Mu rhythm during visuomotor actions, such as golf putting and shooting, is still debated (Chang & Hung, 2020). According to the inhibition hypothesis, the basic assumption of Mu rhythm is that increased Mu power reflects the inhibition of irrelevant motor programming processes. In contrast, decreased Mu power indicates the facilitation of task-relevant motor programming during motor preparation (Klimesch et al., 2007; Pfurtscheller, 2003). In an expert-novice comparison study, Haufler et al. (2000) observed that shooters had higher left central high Mu power (10–11 Hz) than novices. Similarly, elite air pistol shooters had higher left central high Mu event-related synchronization (ERS; 10–12 Hz) during their performances (Del Percio et al., 2009). In a longitudinal study, Kerick et al. (2004) observed that increased Mu in the motor cortex led to improved shooting performance with implicit motor learning. These findings suggest that the inhibition of motor programming processes (i.e., increased Mu power) may avoid undermining the motor program during visuomotor performances. Hence, the enhancement of Mu rhythm may prevent motor programming processing interference during motor preparation, resulting in superior visuomotor skill performance. However, there have been contradictory findings reported in recent studies. Recent data has shown that reduced Mu rhythm plays a crucial role in superior performance in golf putting tasks. For instance, improved motor control (i.e., reduced clubhead acceleration and impact velocity) and performance were associated with reduced Mu in skilled individuals (Cooke et al., 2014; Wang et al., 2020) and during successful performances (Babiloni et al., 2008; Wang et al., 2019). Furthermore, Babiloni et al. (2008) observed that Mu activity positively correlated with error from the hole (cm) in a golf putting task. These findings suggest that functional motor programming during motor preparation is vital for superior performance in golf putting tasks. That is, lower Mu rhythm reflects the facilitation of motor programming when participants need to devote more neural resources to the planning of an action (i.e., movement force and movement direction; Cooke et al., 2015). Thus, decreased Mu rhythm may facilitate task-relevant motor programming processes during visuomotor tasks. Given these conflicting results, examining the function of Mu rhythm during visuomotor tasks could provide a clearer picture of the role of motor programming during visuomotor performance.

These conflicting reports may be due to the use of different variables in the studies, such as the differences in skill levels (e.g., novices vs. experts or low-skilled vs. highly skilled; Cooke et al., 2014; Haufler et al., 2000; Wang et al., 2020) and the complexity of the motor skill (i.e., simple vs complex skills; Wulf & Shea, 2002). Specifically, relative to simple skills, complex skills require (1) the control of more degrees of freedom, (2) increased body movement coordination, and (3) increased effort and information processing from the environment (Magill & Anderson, 2014; Wulf & Shea, 2002). Simple skills, such as indoor shooting, require the performer to process environmental information (i.e., target distance) and require intricate body movements (i.e., hand stability; Del Percio et al., 2009; Lakie, 2010). In contrast, complex skills, such as golf putting, require processing of the environmental context (i.e., target distance and ball path to a target) and the coordination of several intricate body movements (i.e., movement force, direction, and stability; Arsal et al., 2016; Wang et al., 2021). Although both motor skills require object manipulation, different motor representations (simple vs. complex skills) may modulate Mu activity during the motor preparatory processes (Berka et al., 2010).

As these inconsistent findings limit our theoretical and mechanical understanding of the influence of motor programming processes on visuomotor skills (i.e., shooting and golf), the study of control moderators, such as the complexity of the motor skill (Berka et al., 2010) or the difference in skill levels (Cooke et al., 2014; Haufler et al., 2000), may clarify the association between Mu rhythm and the skilled performance of visuomotor tasks. For example, in a longitudinal study, Kerick et al. (2004), who recruited novices, found that increased Mu rhythm was associated with superior visuomotor performance in a shooting task (a simple visuomotor skill). Nevertheless, it is still unclear whether increased Mu rhythm is associated with superior visuomotor performance in complex visuomotor tasks, such as golf putting. If a study of Mu activity in novices performing a golf putting task (a complex visuomotor skill) produces results that are analogous with those of Kerick et al. the difference in skill level, rather than the complexity of the motor skill, may be a potential moderator. In contrast, the complexity of the motor skill may be a potential moderator of Mu activity if Mu activity in these novices is not consistent with the findings in Kerick et al. Hence, recruiting novices to perform a golf putting task will allow us to compare our findings directly with those in Kerick et al. and clarify the relationship between Mu rhythm and visuomotor performance. Additionally, there are very few published experiments that have manipulated Mu rhythm to provide direct causal evidence of its effects (Ros et al., 2014). Thus, manipulating the Mu rhythm of novoices during a golf putting task will help us to confirm the relationship between Mu rhythm and visuomotor performance.

Electroencephalographic-neurofeedback training (EEG-NFT) can be used to manipulate EEG activity directly. EEG-NFT consists of a set of procedures and provides individuals with real-time information about their level of cortical activity via sounds and/or visual displays. Previous studies have shown that a single EEG-NFT session can alter EEG activity and performance. For example, Ros et al. (2014) demonstrated that a single session of EEG-NFT focused on Mu rhythm could enhance implicit motor learning in a serial reaction time task. In sports, Kao et al. (2014) trained three professional golfers to reduce their frontal midline theta (4–7 Hz) power, which is associated with sustained attention during golf putting, by dividing the golfers into three training conditions: a baseline condition, a sitting condition, and a standing condition for a single session of EEG-NFT. Kao et al. (2014) demonstrated that a single session of EEG-NFT could change EEG activity in the resting condition and facilitate golf putting performance. This evidence provides a rationale to use a single session of EEG-NFT in sports training. Using EEG-NFT to manipulate Mu rhythm (i.e., increasing or decreasing Mu rhyhm) in a single session will allow us to directly determine the relationship between Mu rhythm and visuomotor performance and test a potential tool for refining motor programming processes in sports training.

This study aims to clarify the direction of the association between Mu rhythm and skilled performance in visuomotor tasks. To do this, a control moderator (the difference in skill level) and the manipulation of Mu rhythm were utilized during a complex visuomotor task (golf putting). Accordingly, we recruited novice and used EEG-NFT to manipulate Mu rhythm during a golf putting task to understand whether our findings were consistent with Kerick et al. (2004), who recruited novice to perform a shooting task (a simple visuomotor skill). The EEG-NFT protocol was based on Kao et al. (2014) and Ros et al. (2014), who both used a single training session. Given that decreased Mu power has been associated with superior performance in golf putting (Cooke et al., 2014; Wang et al., 2019, 2020), we assumed that decreased Mu power plays an essential role in such performance improvements (Cooke et al., 2014; Wang et al., 2019, 2020). We followed previous protocols (Cooke et al., 2018; Hung & Cheng, 2018) and established three groups to test our working hypothesis (increased Mu rhythm group, IMG; decreased Mu rhythm group, DMG; sham group, SG). We hypothesized that the DMG would display greater performance improvements than the IMG and SG groups after EEG-NFT. Furthermore, in terms of Mu activity, we hypothesized that the DMG would exhibit significantly decreased Mu power after EEG-NFT and the IMG would exhibit significantly increased Mu power following EEG-NFT.

## Method

### Participants

Power analysis for the repeated measures multivariate analysis of variance (MANOVA) was performed for a sample size calculation using G*Power to determine the minimal detectable effect (Faul et al., 2007). Following previous studies using a similar research design (Ring et al., 2015), we used the following values: *α* = 0.05, power = 0.80, effect size = 0.70 (corresponding to *η*_*p*_^2^ = 0.33), number of groups (IMG, DMG, and SG) = 3, and number of measurements (pre-post measurements × electrode sites) = 8. The resulting minimum sample size was N = 26. We recruited 30 novices because of the potential for power analysis biases, which has been highlighted in the neuroscience field (Albers & Lakens, 2018; Algermissen & Mehler, 2018). They were assigned to the IMG (5 females, 5 males; mean age = 27.40 ± 6.83), DMG (5 females, 5 males; mean age = 29.00 ± 8.43), and SG (5 females, 5 males; mean age = 25.90 ± 5.44). All of the recruited participants met the following selection criteria: (1) no history of psychiatric or neurological disease; (2) right-handed (Oldfield, 1971); (3) not taking medicine affecting the central nervous system or brain; (4) normal or corrected-to-normal vision; and (5) normal visual attention. Additionally, all of the participants provided informed consent before taking part in the experiment.

### Measures

#### Golf putting Task

The participants used a standard putter for regular-sized golf balls (diameter = 4.27 cm) to putt balls towards a target from a 3-m distance on an artificial putting green (4 m × 9 m). Both before and after the EEG-NFT intervention, the participants performed 20 putts (i.e., pretest–posttest). For each trial, backswing movement was detected by an infrared sensor as an event marker. The definition of the motor preparation period was that specified by Wang et al. (2020) who defined it as the period between placing the putter behind the ball and initiating the backswing.

#### Subjective Stress Level

To counter the confounding effects of stress, subjective stress level was assessed with a perceived stress score rated on an 11-point Likert scale, ranging from 0 (no stress at all) to 11 (highest stress level; di Fronso et al., 2016) during the golf putting task both before and after NFT.

#### Subjective Psychological State (Attentional Control Level)

In addition to the effects of NFT on behavioral outcomes, self-evaluation was required to examine the effects of NFT on psychological states, such as the level of attentional control of the action (Hung & Cheng, 2018). Given that 8–13 Hz at the central cortex (Cz) has been associated with motor programming (i.e., motor control), participants were asked to rate their level of attentional control of the action on a 11-point Likert scale, ranging from 0 (not at all) to 11 (maximum possible; di Fronso et al., 2016). The participants were asked to report their attentional control levels on the action during the golf putting task both before and after NFT.

### Instrumentation

#### Vicon Motion Systems

We used a motion capture system (Vicon Motion Systems, Oxford, UK) to record putting performance. Specifically, six T10 charge-coupled device cameras tracked the ball rolling and stopping. The data were recorded with a spatial resolution of approximately 0.25 mm and a temporal resolution of 200 Hz.

#### EEG

Electrodes were placed in accordance with the international 10–10 system, with 64 electrode sites recorded in total. The electrical reference was located on the left and right ear mastoids (M1, M2), and the ground electrode was located at the anterior frontal zone position (AFz; Jurcak et al., 2007). Vertical and horizontal electrooculograms (HEOL, HEOR, VEOU, and VEOL) were recorded with bipolar configurations located superior and inferior to the left eye and on the left and right orbital canthi. The eego system (ANT Neuro, Germany) was used with a bandpass filter from 1 to 100 Hz and a 50 Hz Notch filter. The eego software was used to collect data with a sampling frequency of 500 Hz. Electrode impedance was kept below 10 kΩ. For the Mu rhythm, 8–13 Hz at Cz was extracted (Wang et al., 2019, 2020).

#### Neurofeedback Recording

Neurofeedback training was completed with the BioTrace + software (MindMedia, NeXus-10, the Netherlands). Signals were acquired using a DC-coupled EEG amplifier with a 24-bit A/D converter to extract the Mu rhythm. The amplitude of the Mu rhythm was transformed into an audio-feedback tone using acoustic bass.

### Procedures

We used a stratified random control experimental design by gender to divide the population into three subgroups (IMG, DMG, and SG). We used a pretest–posttest design for a single training session (Kao et al., 2014; Ros et al., 2014). Thus, our study included three groups as a between-subject factor and a pre-posttest measurement as a within-subject factor. We asked the participants not to consume any food or beverages containing alcohol or caffeine 24 h prior to the testing day. On the testing day, we (a) asked the participants to provide a negative coronavirus (COVID-19) test, (b) explained the nature of the study, (c) asked the participants to sign an informed consent form, and then asked them to (d) put on the Lycra electrode cap, (e) watch a putting video (15 s) without any golf instruction, (f) perform a warm-up using ten balls, with the goal of putting the golf ball as accurately as possible, (g) report their attentional control and stress levels, (h) perform 20 putts (pretest) (h) complete the EEG-NFT intervention, (i) report their attentional control and stress levels again, and (j) perform 20 putts (posttest). The experiment lasted approximately 2.5 h in total.

#### Neurofeedback Training Protocol

Cortical activity was recorded from the Cz site on the EEG cap. The reference and ground electrodes were attached to the left and right ear mastoids, respectively. The EEG-NFT procedure for Mu lasted approximately 30–45 min. Two training stages (i.e., pre-EEG-NFT, acquisition) were carried out. In the pre-EEG-NFT stage, the participants were asked to perform ten putts to warm up. Next, the average Mu amplitude over the ten putts was defined as the training criteria (training baseline) for each participant. We set ± 20% of the baseline as a training target for the IMG and DMG (Kao et al., 2014). We then asked all participants to develop their own strategies for implicit learning (Ros et al., 2014) associated with the golf putting task to maintain their Mu amplitude within the criteria via visual feedback or auditory feedback. Visual feedback is visual output from a system, such as a computer game, that allows a participant to interact better with the system. For example, participants could see visual displays of the Mu signals on a screen. Auditory feedback is auditory output from a system, such as sound effects. For example, participants could hear feedback when they reached a prescribed level of Mu activity. The instructions provided to all participants were as follows: “The computer will play a tone that is linked to your brain activity. When you reach a prescribed level of brain activity, the tone will sound. This means that you are in an optimal mental state, and you need to remember the feeling that you experience when you receive the feedback.

However, for the SC group, we used a random feedback tone frequency during the training trials. To guarantee the randomized feedback tone, researchers randomly played the feedback tone using random.org. To ensure that EEG-NFT learning could be achieved, the participants were required to meet a successful training ratio of 70% (Gruzelier, 2014) in a single training trial (40 s), which was defined as the amount of time that the participant successfully entered the training threshold during the motor preparation period. If participants did not achieve the training ratio of 70%, the Mu power baseline would be increased/decreased by 10% until the training ratio of 70% was achieved.

In the acquisition stage, the participants were exposed to two different conditions (i.e., sitting and standing) to progressively simulate real-life putting conditions (Kao et al., 2014). To enhance the EEG-NFT efficacy, progressive adjustments of the training threshold of ± 10% in the sitting condition and ± 20% in the standing condition were used. Additionally, the successful training ratio was set to 80%. That is, a higher ratio indicated better control of the EEG. For the sitting condition, participants were asked to sit 60 cm in front of a computer monitor. The average successful training ratio was above 80% for three consecutive trials, with at least six blocks of audiovisual feedback. When this was achieved, the participants were allowed to progress to the standing condition. For the standing condition, the participants were asked to maintain their preputt posture while holding a putter. Visual feedback was removed to enable participants to engage in a real-life preputt routine. The training protocol was the same as for the sitting condition, and the participant’s average successful training ratio had to exceed 80% during three consecutive trials of at least six blocks before they could progress to the posttask assessments.

### Data Analysis

#### Behavioral Data

To measure performance outcomes, we calculated putting accuracy using the pre-posttest mean radial error (MRE; Frank et al., 2016). MRE is defined as the average distance (mm) of each subject’s putt outcomes from the center of the target.

#### EEG Data

The EEG data were preprocessed using EEGLAB functions (Delorme & Makeig, 2004) and custom scripts written in MATLAB (MathWorks, U.S.A.). The EEG preprocessing steps consisted of (1) re-referencing the EEG data to the averaged mastoids (A1, A2); (2) setting the bandpass filter from 1 Hz (low-pass) to 30 Hz (high-pass) using a basic finite infinite response (FIR) filter; (3) extracting epochs from the − 3,000 to 1,000 ms time window before putting; (4) removing channels with bad signals; (5) rejecting gross artifacts (amplitudes exceeding ± 100 µV) to eliminate any potential biological artifacts (e.g., muscle activation artifacts; Wang et al., 2020), resulting in 49 trials being rejected pretest (IMG = 1 ± 0.031 trials, DMG = 5 ± 0.70 trials, SG = 13 ± 2.54 trials) and posttest (IMG = 2 ± 0.042 trials, DMG = 4 ± 0.69 trials, SG = 24 ± 4.16 trials); (6) running independent component analysis (ICA; Runica Infomax algorithm; Makeig et al., 1996) to identify and remove components arising from blinks, eye movements, and other non-neural activity; (7) interpolating channels with bad signals; (8) dividing the clean signals into 2-s epochs (− 2,000 to 0 ms before putting); and (9) calculating the 8–13 Hz power spectrum using the Welch estimation method (Hanning windowing function; Welch, 1967).

The pretest trial counts for IMG, DMG, and SG were 19.90 ± 0.31, 19.50 ± 0.70, and 18.7 ± 2.54 trials, respectively. Posttest trial counts were 19.80 ± 0.42, 19.60 ± 0.69, and 17.60 ± 4.16 trials, respectively. A one-way analysis of variance (ANOVA) was performed to address concerns that differences in the number of trial counts between the groups could confound the results. The results showed no significant differences between the groups both pretest (*p* = 0.223) and posttest (*p* = 0.104). Thus, the unequal number of trials did not affect our findings. For brevity of reporting, only the results from the key Cz electrode, and those in its immediate surroundings (i.e., C3 and C4) are presented. We selected these electrodes because they roughly overlie the frontal lobe, which consists of primary motor cortex, the premotor cortex, and the supplementary motor areas that are related to movement programming processes, all of which have been implicated in previous EEG-based golf-putting research (Babiloni et al., 2008; Cooke et al., 2014, 2015; Wang et al., 2019, 2020).

#### Source Analysis (sLORETA)

To explore any training-induced changes in the EEG recordings, standardized low resolution brain electromagnetic tomography (sLORETA) can be used to identify the sources of specific cortical rhythms (Sekihara et al., 2005; Wagner et al., 2004). sLORETA (The KEY Institute for Brain-Mind Research, Zurich; Pascual-Marqui & Roberto Domingo, 2002) was applied to the three-dimensional cortical distributions of the averaged pretest and posttest amplitudes separately. The cortical and hippocampal gray matter images represent 6,239 voxels, with a spatial resolution of 5 mm. The digitized structural MRI template was selected from a realistic head model (Fuchs et al., 2002) with the probabilistic MNI152 template (Mazziotta et al., 2001).

### Statistical Analysis

The behavior data and EEG data was exported from the motion capture system (Vicon Motion Systems), the eego system (ANT Neuro), and the NeXus-10 system (MindMedia). We further used SPSS 22 software for statistical analysis. Separated Mixed-design ANOVA and MANOVA were performed on our measures (more details in Results section). The alpha level was set at 0.05.

## Results

### Age

A one-way ANOVA was used for the age distributions of the three groups (IMG, DMG, and SG). Our data demonstrated that there was no significant effect of age on the results, *F*(2, 27) = 0.489, *p* = 0.619.

### Putting Performance

**Fig. 1:**
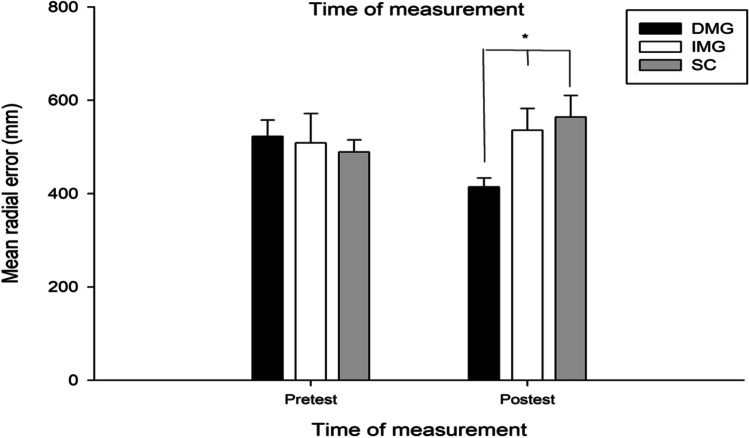
Mean radial error in mm from pretest to posttest. *Note*. Mean radial error in mm is shown for the putting accuracy from pretest to posttest in three groups. Error bars represent standard errors. *p < 0.05

To determine the effect of EEG-NFT on golf putting performance, we ran a 3 (groups: IMG, DMG, SG) × 2 (time: pretest, posttest) repeated measures ANOVA of the MREs. A repeated measures ANOVA revealed a significant interaction effect between time and group, *F*(2, 27) = 4.310, *p* = 0.024, and *η*_*P*_^*2*^ = 0.242. Follow-up analyses indicated that the DMG had a significantly lower MRE than the IMG (*p* = 0.038, *d* =  − 1.075) and the SG (*p* = 0.012, *d* =  − 1.344) after EEG-NFT. Additionally, only the DMG exhibited performance improvements after EEG-NFT (*p* = 0.006, *d* =  − 0.985, Fig. 1).

### Subjective Psychological State (Attentional Control Level)

To examine the causal relationship between brain activity and psychological state, we ran a 3 (groups) × 2 (time) repeated measures ANOVA of the self-evaluation data (e.g., the level of attentional control). A repeated measures ANOVA revealed a significant interaction effect between time and group, *F*(2, 27) = 6.247*, p* = 0.006*,* and *η*_*P*_^*2*^ = 0.316*.* Follow-up analyses indicated that the DMG had a significantly higher level of action control than the IMG *(p* = 0.018*, d* =  − 1.013*)* and the SC *(p* = 0.011*, d* =  − 1.306*)* following EEG-NFT. Additionally, only the DMG had a significantly increased level of action control after EEG-NFT *(p* = 0.003*, d* = 0.943*).*

### Putting-State EEG

#### Brain Regions

To examine the topographical specificity of 8–13 Hz at the Cz, a 3 (groups) × 2 (time) repeated measures MANOVA was carried out for the 8–13 Hz power at Fz, Cz, Pz, and Oz sites. As can be seen in Table 1, a significant interaction was seen between group and time, *F*(8, 48) = 2.580, *p* = 0.020, Wilks’ lambda = 0.489, *η*_*P*_^*2*^ = 0.301, and power = 0.871. Univariate analyses showed a significant interaction between group and time at Cz, *F*(2, 27) = 4.763, *p* = 0.017, and *η*_*p*_^2^ = 0.261. Post hoc analyses indicated that the DMG exhibited significantly lower 8–13 Hz power at Cz than the IMG (*p* = 0.005, *d* = 1.47) following EEG-NFT. Interestingly, no significant difference was seen in 8–13 Hz power at Cz between the DMG and SG (*p* = 0.097, *d* = 0.748) after EEG-NFT. Importantly, 8–13 Hz power at Cz was only significantly decreased in the DMG (*p* < 0.001, *d* = –1.255) after EEG-NFT.

**Table 1:** Results of a 3 (groups) × 2 (time) repeated measures MANOVA for the 8–13 Hz power at Fz, Cz, Pz, and Oz sites

			Value	*F*	*Hypothesis df*	*Error df*	*p*		*η*_*p*_^2^	*Power*		
Wilks’ lambda			0.489	2.580	8	48	0.020		0.301	0.871		
Univariate Tests	Fz			Cz			Pz			Oz		
	*df*	*F*	*p*	*df*	*F*	*p*	*df*	*F*	*p*	*df*	*F*	*p*
Groups*Time	2,27	1.955	0.161	2,27	4.763	0.017	2,27	0.281	0.747	2,27	1.385	0.267

*N* = *30. df* is the degrees of freedom. *F* is the F-value. *p* is the *p*-value

#### Frequency Bands

To examine frequency specificity, we ran a 3 (groups) × 2 (time) repeated measures MANOVA of 4–7 Hz, 8–13 Hz, and 14–20 Hz power at Cz. As shown in Table 2, we confirmed that there was a significant group and time interaction, *F*(6, 50) = 2.788, *p* = 0.020, Wilks’ lambda = 0.562, *η*_*P*_^*2*^ = 0.251, and power = 0.837. As expected, a significant interaction between group and time in the 8–13 Hz in central region was observed, *F*(2, 27) = 4.763, *p* = 0.017, and *η*_*p*_^2^ = 0.261. Post hoc analyses indicated the same results as those in the Brain regions analysis.

**Table 2:** Results of a 3 (groups) × 2 (time) repeated measures MANOVA of 4–7 Hz, 8–13 Hz, and 14–20 Hz power at Cz

	Value	*F*	*Hypothesis df*	*Error df*	*p*	*η*_*p*_^2^	*Power*		
Wilks’ lambda	0.562	2.788	6	50	0.020	0.251	0.837		
Univariate Tests	4–7 Hz			8–13 Hz			14–20 Hz		
	*df*	*F*	*p*	*df*	*F*	*p*	*df*	*F*	*p*
Groups*Time	2,27	0.536	0.591	2,27	4.763	0.017	2,27	0.262	0.771

*N* = *30. df* is the degrees of freedom. *F* is the F-value. *p* is the *p*-value

#### sLORETA of the Decreased Mu Rhythm Group

To explore any training-induced changes in the EEG recordings, pretest and posttest sLORETA images were compared using a paired t-test. Statistical non-parametric mapping of the sLORETA images were performed with sLORETA’s built-in voxel-wise randomization tests (5,000 permutations) and by using a log-F-ratio statistic with a threshold of *p* < 0.05, corrected for multiple comparisons (Nichols & Holmes, 2002).

**Fig. 2:**
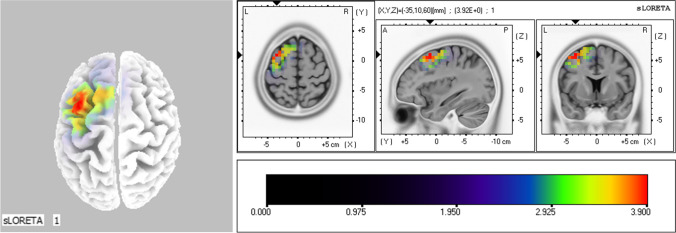
8–13 Hz (Pretest − posttest). *Note*. Results of the sLORETA analysis of 8–13 Hz power (contrast: pretest − posttest) during motor preparation (− 2,000 to 0 ms). Images were obtained after statistical nonparametric mapping. Yellow colors indicate voxels with siginiciantly increased power at 8–13 Hz

As only the DMG experienced significant training-induced changes in 8–13 Hz power, we subsequently focused on this group. The middle frontal gyrus (MFG) exhibited significantly lower 8–13 Hz power in the DMG, reflecting enhanced activation following EEG-NFT (see Fig. 2 and Table 3). The maximum difference was located in the MFG: threshold value *(T)* = 3.92, *p* < 0.05 *(T* = 3.90; see Fig. 2*).*

**Table 3:** Brain areas that experienced a stronger activation after EEG-NFT in the DMG (compared to pretest values)

Location		Brodmann area	MNI coordinates			*t*-value
			*X*	*Y*	*Z*	
Frontal Lobe	Middle Frontal Gyrus	6	− 35	10	60	3.92

Brain areas with statistical differences (*t* > 3.901, *p* < 0.05) are shown. MNI coordinates and *t-*values show the maximum value for each location. Coordinates are given in millimeters, with an origin of the MFG. For *x*, negative values represent left, positive values represent right. For *y*, negative values represent posterior, positive values represent anterior. For *z*, negative values represent inferior, positive values represent superior

### Neurofeedback Training

To determine the learning effect of EEG-NFT, we compared 8–13 Hz power at Cz during the first and last block during the training period. A 2 (groups) × 2 (block: first and last block) repeated measures ANOVA was performed. Analyses revealed a significant interaction effect between time and group, *F(*1, 18*)* = 40.809, *p* < 0.001*,* and *η*_*P*_^*2*^ = 0.694*.* Follow-up post hoc analyses indicated that the DMG had significantly decreased 8–13 Hz power at Cz from the first block to the last block (*p* < 0.001*).* In contrast, power was significantly increased in the IMG (*p* = 0.005)*.* Thus, EEG-NFT was effective in changing the targeted brain activity in novice golfers.

### Correlation Between Changes in Mu Rhythm and Performance

To further test the correlation between Mu rhythm and performance, we performed a correlational analysis between the percentage change in Mu activity and the percentage change in performance from pretest to posttest. A Pearson's correlation analysis revealed that the percentage change in Mu activity was not significantly correlated with the percentage change in performance (*r* = 0.182*, p* = 0.168*, N* = 30).

### Control Analysis

To counter the confounding effects of stress, we ran a 3 (groups) × 2 (time) repeated measures ANOVA of the subjective self-reported stress levels. Pre- and posttest self-reported subjective stress levels were compared both between and within subjects. A repeated measures ANOVA revealed no significant interaction effect between time and group (*p* = 0.306), nor any effect of time (*p* = 0.211) specifically, suggesting that stress levels did not significantly change during the course of the experiment and likely did not affect the results.

## Discussion

This study aimed to determine the direction of the association between Mu rhythm and skilled performance of visuomotor tasks. To achieve this, we manipulated Mu power using EEG-NFT in novice golfers. We compared Mu power and golf putting performance in the DMG, IMG, and SG. Our main finding was that following EEG-NFT, the DMG experienced significantly decreased Mu power, while the IMG did not display significantly increased Mu power. Furthermore, the DMG had significantly increased perceived control of action and improved performance following EEG-NFT. These findings partially support a causal relationship between Mu power and golf putting performance, improving our understanding of motor programming processes in skilled sports.

Regarding the effects of EEG-NFT on Mu rhythm, we found that the DMG experienced significantly decreased Mu power following a single NFT session. This finding not only corroborates Ros et al. (2014), who demonstrated that a single session of Mu rhythm EEG-NFT could enhance implicit motor learning in a serial reaction time task, but also extends upon their findings by showing that a single session of NFT can decrease Mu power during a complex visuomotor task (i.e., golf putting). Moreover, the current study is the first to use the Mu protocol to investigate the effectiveness of EEG-NFT on sport performance. Nevertheless, we did not observe significant increases in the IMG Mu power from pretest to posttest. This might be because the EEG-NFT approach used in this study used implicit verbal instruction. We asked participants to develop their own strategies for controlling Mu power during the intervention. Such abstract verbal instructions did not include explicit instructions for how to induce the targeted neural activities in the specific region (i.e., increasing Mu power). Given that the performer must process environmental context (i.e., ball path and target distance) and coordinate intricate body movements (i.e., movement force, direction, and stability) during motor preparation in a golf putting task, it might be easier to decrease (i.e., facilitating the motor programming) than increase (i.e., inhibition of motor programming) Mu power. These abstract verbal instructions increase the risk of non-learning within the limited duration of the experiment (Muñoz-Moldes & Cleeremans, 2020), resulting in slower initial learning phases (Kao et al., 2014) as novice golfers may be uncertain how to inhibit their motor programming during the EEG-NFT. Future studies should consider using explicit verbal instructions for increasing Mu power during EEG-NFT, as researchers have shown that explicit verbal instructions can help participants to improve and achieve better learning outcomes during the initial learning phases of EEG-NFT (DeCharms et al., 2005; Scheinost et al., 2013; Zotev et al., 2013).

Turning to the effect of Mu EEG-NFT on subjective psychological state (the level of attentional control of the action) and performance, we observed that only the DMG experienced a significantly increased perceived action control level (i.e., a higher level of attentional control) and improvements in putting performance after EEG-NFT. Our findings are consistent with Ros et al. (2014), who observed that decreasing Mu led to improved motor skills. Additionally, we built upon the findings of Ros et al. (2014) by showing that such performance improvements are associated with increased action control levels (a subjective psychological state). Increased control levels may reflect improved resource allocation during motor programming. Neurophysiological evidence has suggested that 8–13 Hz at Cz (i.e., Mu rhythm) reflects the allocation of cognitive resources to response motor programming (Pineda, 2005) during the execution of goal-directed actions and observations (Cannon et al., 2014). Studies have shown that successful movement identification (Denis et al., 2017) and execution (Fox et al., 2016) are associated with reduced Mu power. Similarly, in sports studies, decreased Mu power has been associated with increased golf putting performance (Wang et al., 2020), successful putting (i.e., the ball going in the hole; Babiloni et al., 2008; Cooke et al., 2014; Wang et al., 2019), and corrective action (Cooke et al., 2015). These findings suggest that lower Mu power reflects greater cognitive resource allocation to response motor programming, resulting in adaptive motor control and increased action control levels during complex visuomotor tasks (Klimesch et al., 2007; Pfurtscheller, 2003), and thus performance improvement. To more precisely locate the cortical region associated with the effects of EEG-NFT, we used source analysis (sLORETA). We found that the MFG was significantly activated in the DMG following EEG-NFT. The MFG in the frontal lobe has been associated with monitoring actions (Milton et al., 2007) and its activation correlates with motor control (constant swing speed and angle in golf; Kim et al., 2015). Hence, a higher level of attentional control on the action may reflect increased action monitoring or motor control (e.g., constant movement) which benefits performance in a complex visuomotor task. These findings not only support our hypothesis by showing that decreasing Mu power can improve complex visuomotor performance, but also provide evidence for a causal relationship between Mu, subjective psychological state (the level of attentional control of the action), and sports performance. Nevertheless, no significant correlation between the percentage change in Mu activity and the percentage change in performance was observed. This result did not support the hypothesis that the largest behavioral change should be correlated with the largest change in the targeted brain activity (Cooke et al., 2018). That is, the effect of Mu activity during a skilled motor performance may be more complex than initially believed. Performance improvement may be associated with individual zones of optimal function, with reduced performance improvement or performance deterioration being associated with either very low or very high percentage changes in the Mu activity (i.e., outside the optimal zone). We encourage future studies to examine this hypothesis.

Overall, we conclude that the complexity of the motor skill being performed may be a potential moderator of Mu rhythm and visuomotor performance, as our results were not analogous with Kerick et al. (2004) who reported that increased Mu rhythm in novices improved performance in a simple visuomotor task. Additionally, our research supports the hypothesis that decreased Mu power represents facilitation of task-relevant motor programming processes during motor preparation (Klimesch et al., 2007; Pfurtscheller, 2003). We provide a possible link between Mu rhythm, subjective psychological state (the level of attentional control of the action), and visuomotor performance. We suggest that greater cognitive resource allocation to response motor programming leads to adaptive motor control during the execution of complex visuomotor tasks. Importantly, we provide an effective EEG-NFT protocol that can effectively decrease Mu power, increase action control levels (a subjective psychological state), and improve golf putting performance in novice golfers.

Our study control first confirmed the frequency specificity (i.e., 8–13 Hz) and topographical specificity (i.e., central region) of the EEG-NFT. Our data showed that the pre- and posttest powers of two neighboring frequency bands (i.e., 4–7 Hz, 14–20 Hz) were not significantly different at the Cz in the DMG. Additionally, pre- and posttest 8–13 Hz power at three other electrodes (i.e., Fz, Pz, and Oz) were not significantly different in the DMG. These findings indicate that we successfully manipulated Mu power using EEG-NFT, and that Mu rhythm plays an essential role in superior performance of a complex visuomotor task. The use of a sham control reduces the likelihood of a placebo effect being responsible for the observed effects of EEG-NFT.

Future research should address the limitations of our study. First, it is worth considering the generalizability of our results. Given that we recruited novices to our study, it may be difficult to generalize to amateurs and experts. Differences in motor skill levels can lead to significant differences in neural characteristics during performance (Gong et al., 2021; Wang et al., 2020). We suggest that EEG-NFT should thus consider participants’ motor learning stages. Thus, future EEG-NFT studies could replicate our study on amateurs or experts. Second, the IMG did not exhibit significantly increased Mu power in EEG analysis following a single NFT session, although Mu power was significantly increased in neurofeedback analysis, potentially due to the implicit nature of the instructions provided. Given that verbal instructions relating to cognitive strategies have a strong relationship with brain function during EEG-NFT learning (Chen et al., 2022; DeCharms et al., 2005), future studies should consider providing verbal instructions for cognitive strategies that can increase Mu power. This will also help researchers to determine whether increased Mu power can also improve performance in a complex visuomotor task.

In terms of the implications of this study for coaches and athletes, we support the implementation of NFT training and further confirm the feasibility of EEG tuning within a single training session (Kao et al., 2014; Ros et al., 2014). Given that we found that decreased Mu power is associated with increased action control levels, resulting in superior motor performance after a single EEG-NFT training session, we suggest that our training protocol can improve motor performance in other complex visuomotor tasks (e.g., archery and basketball). Additionally, this evidence aligns with previous work in which decreased Mu power has been associated with adaptive motor control during a complex visuomotor task (Babiloni et al., 2008; Cooke et al., 2014, 2015; Wang et al., 2019, 2020).

In conclusion, the complexity of the motor skill, rather than differences in skill level, may be a potential moderator of Mu rhythm and visuomotor performance of skills. Additionally, we found that EEG-NFT is a valuable tool for decreasing Mu rhythm, which is associated with increased action control levels and golf putting performance enhancement. We suggest that devoting neural resources to response motor programming leads to adaptive motor control during the performance of a complex visuomotor task. These findings partially support the causal relationship between Mu power, subjective psychological state (the level of attentional control of the action), and sports performance improvements achieved using EEG-NFT.

## Data Availability

The datasets generated during and/or analysed during the current study are available from the corresponding author on reasonable request.
